# The population characteristics of the main leukocyte subsets and their association with chronic diseases in a community-dwelling population: a cross-sectional study

**DOI:** 10.1017/S1463423621000153

**Published:** 2021-05-07

**Authors:** Wangyang Chen, Jixi Wang, Bintao Ye, Jun Zhou, Weibo Wang

**Affiliations:** 1 Department of Preventive Medicine, College of Medicine, Taizhou University, Taizhou, Zhejiang Province, China; 2 Department of Anatomy, College of Medicine, Jiaxing University, Jiaxing, Zhejiang Province, China; 3 Department of General Practice, BaiYun Community Health Service Center, Taizhou, Zhejiang Province, China

**Keywords:** chronic disease, leukocytes, lymphocytes, monocytes, neutrophils

## Abstract

**Aim:**

To analyse the characteristics of the main leukocyte subsets and elucidate their distributions amongst the natural population. We wanted to determine whether leukocyte subsets are potential biomarkers to evaluate the risk of common chronic diseases.

**Background:**

The peripheral blood leukocyte count is a routine exam performed to detect pathogen infections. Recently, subsets of white blood cells and their homeostasis have shown strong associations with some chronic diseases. Therefore, studies aiming to discover whether the distribution of leukocyte counts and its subsets are useful for predicting health conditions are worthwhile.

**Methods:**

This cross-sectional study analysed 10 564 residents from the basic public health service project of the Health Checkup Program performed by the BaiYun Community Health Service Center. Data on demographic information, physical measurements, medical history, and routine blood examination parameters were collected using questionnaires and health check-ups. Restricted cubic spline incorporated into logistic regression analysis was performed to evaluate the association between subsets of leukocytes and common chronic diseases.

**Findings:**

The counts of leukocytes and their subsets in males were higher than those in females amongst all age groups, yet the percentages of lymphocytes and neutrophils did not present sex-specific differences. A low lymphocyte count and percentage were associated with old age. The neutrophil-to-lymphocyte ratio (NLR) in patients with hypertension was higher than that in the non-hypertensive population. The risk of NLR in the top quartiles was 1.17-fold higher than that in people in the lowest quartiles.

**Conclusions:**

The distributions of the white blood cell count and percentage were associated with age, sex, and body mass index (BMI). In addition to the immune barrier for pathogens, the NLR or monocyte-to-lymphocyte ratio (MLR) may be potentially used to indicate the risk of some chronic non-communicable diseases. Homeostasis of subsets of leukocytes may be an important biomarker for body health conditions.

## Introduction

The peripheral blood leukocyte count has been considered to play important roles in the cellular-mediated inflammatory response. Recent studies have provided extensive information on the robust relationship of leukocytes, especially their subsets of cells, with chronic diseases such as stroke, diabetes mellitus, hypertension (Lee *et al.*, 2017; Demirdal & Sen, 2018). Peripheral lymphocytes, neutrophils, monocytes, eosinophils, and basophils are the main subsets of white blood cells. The neutrophil-to-lymphocyte ratio (NLR) and monocyte-to-lymphocyte ratio (MLR) are proxy markers for systemic inflammation (Pinato *et al.*, 2012). Some studies discovered correlations between many inflammation-associated diseases such as intracranial atherosclerosis (Nam *et al.*, 2018), type 2 diabetes (Mertoglu & Gunay, 2017), and hypertension (Jhuang *et al.*, 2019), and the NLR or MLR. Furthermore, leukocytes have been established to correlate with high blood pressure (Rodriguez-Iturbe *et al.*, 2017). Given the strong association of leukocytes with health conditions, mechanistic studies on the relationship between them have been gradually reported.

Leukocyte homeostasis and the ratio of each subset of cells or component modulation are complex. Research has suggested that one neutrophil subset (polymorphonuclear myeloid-derived suppressor cells) exerts inhibitory effects on lymphocytes, specifically the inhibition of lymphocyte proliferation (Lang *et al.*, 2018). The ratio of neutrophils to lymphocytes positively correlates with age in the healthy population (Li *et al.*, 2015). The lymphocyte number, neutrophil count, and platelet count were found to have significantly positive linear correlations with obesity, which is a confirmed risk factor for some chronic diseases (Furuncuoglu *et al.*, 2016). As many factors contribute to the fluctuation of the leukocyte count, a systematic evaluation conducted after balancing the potential risk factors is more precise for indicating health problems.

Because a complete blood count is easy to analyse and effectively predict systematic homeostasis, it is considered as a routine examination for regular health check-ups. In the current study, we wanted to explore the trends in age-, sex-, weight-, and disease-specific distributions of haematological immune cells in a community-dwelling population. Furthermore, some factors were anticipated to prevent or treat chronic diseases such as hypertension and type 2 diabetes by confirming the effect of abnormal leukocyte subset distributions on risk.

## Materials and methods

### Study design and participants

Data analysed in this study were collected from the basic public health service project of the Health Checkup Program performed by the BaiYun Community Health Service Center, Taizhou city from May 2015 to November 2019. A total of 10 564 participants residing in the BaiYun community with available data from the first routine check-up, including routine blood examination, anthropometric measures, and blood pressure were selected for this investigation. Information on the medical history of hypertension, diabetes, stroke, and demographics was obtained using questionnaires administered by nurses. One-hundred and eighty-four individuals were excluded from this analysis due to the absence of leukocyte subset count records. This study protocol and procedure were approved according to the ethical standards of the Declaration of Helsinki, and all participants provided informed consent.

### Blood parameters

Fasting blood samples were collected from residents at BaiYun Community Health Service Center, and complete blood count measurements were performed immediately using standardised procedures. Blood parameters, including the leukocyte count and counts of its main subtypes (neutrophils, lymphocytes, and monocytes) and percentages were determined using an automatic blood cell analyzer (BC-5380, Shenzhen Mindray Bio-Medical Electronics Co., Ltd.).

### Calculation of all kinds of ratio

Body mass index (BMI, kg/m^2^) was calculated by dividing the participant’s weight (kilogram) by height (metre) squared. BMI was categorised into four groups based on the Chinese reference standard: underweight (<18.5 kg/m^2^), normal weight (18.5–24.0 kg/m^2^), overweight (24.0–30.0 kg/m^2^), and obese (≥30.0 kg/m^2^). The NLR and MLR were calculated based on absolute peripheral neutrophil and monocyte counts, respectively, divided by lymphocyte counts in the blood.

### Definitions of hypertension, diabetes, and stroke

Hypertension was defined as systolic blood pressure ≥140 mmHg or diastolic blood pressure ≥90 mmHg, a previous diagnosis of hypertension, and the use of any antihypertensive drugs. Type 2 diabetes mellitus was defined as fasting blood glucose levels ≥7.0 mmol/L, a previous diagnosis of diabetes mellitus or the use of any hypoglycaemic drugs. Stoke includes ischaemic and haemorrhagic stroke subtypes and was distinguished according to the presenting symptoms, such as awakening with or experiencing the abrupt onset of focal neurological deficits and non-contrast head computed tomography imaging indicating haemorrhage or ischaemic change in the medical records.

In this study, chronic disease refers to the hypertension, type 2 diabetes mellitus, and stroke listed above.

### Statistical analysis

Continuous data are presented as the means ± standard deviations for quantitative variables, and categorical variables are presented as percentages. Differences in normal variables between groups were tested using Student’s *t*-test, and non-normal data were tested using the Mann–Whitney *U* test. The chi-square test was applied to analyse categorical data. The main effects of cell counts and percentages were tested using the general linear model. The NLR and MLR values were divided into quartiles as 25%, 50%, and 75% of each marker, and the first quartile was used as a reference in the multivariable analysis. The potential of blood immune cell parameters to predict the risk of target chronic diseases was estimated using a logistic regression model. Restricted cubic spline regression was used to explore the potential dose–response relationship between chronic diseases and NLR, MLR.

Statistical analyses were performed using SPSS version 24.0 (IBM Corp.), and R version 4.0.3. Two-sided *P*-values <0.05 were regarded as indicating statistical significance.

## Results

### Characteristics of the participants

**Figure 1.**
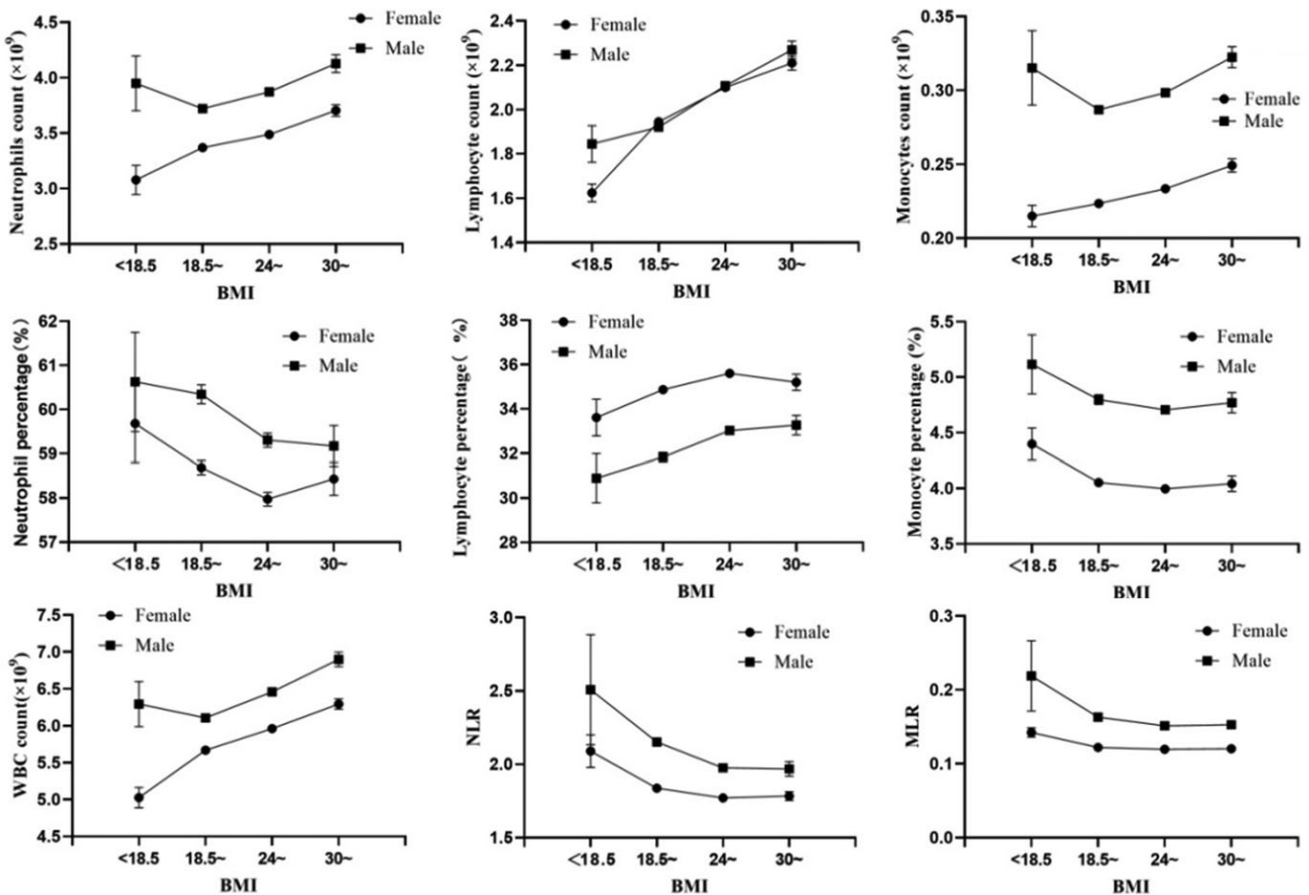
Principle peripheral blood immune cells distribution in different BMI groups.

The majority of participants were female, accounting for 54.87% of the 10 564 subjects. Six-thousand and six-hundred one (6601) subjects were older people aged ≥60 years. Approximately 58.52% of participants were overweight (BMI > 24 kg/m^2^), and 7.05% of them were obese (BMI > 30 kg/m^2^). The trend for the mean BMI in different age groups was not the same between males and females. In the group aged less than 60 years, men had a higher BMI than women. However, the values were similar in the old population (Figure 1). The prevalence of hypertension, diabetes, and stroke was 47.34%, 16.25%, and 0.89%, respectively (Table 1).

### Association of peripheral blood white blood cell subsets with BMI

A significant difference in the types of peripheral blood immune cells was observed between males (6.36 ± 1.63 × 10^9^) and females (5.83 ± 1.46 × 10^9^) (*t* = 17.407, *P* < 0.001). The total white blood cell (WBC) count and principal component cell counts, such as neutrophil, lymphocyte, and monocyte counts, increased with the BMI. The percentages of neutrophils and monocytes decreased with increasing BMI, but the lymphocyte percentage was positively associated with BMI. More interestingly, lymphocyte counts were not different between females and males (*F* = 0.336, *P* = 0.562), and lymphocyte percentages were higher in females than in males after adjusting for the variables age and BMI in the general linear model (*F* = 298.88, *P* < 0.001). The NLR was lowest in the largest BMI group, regardless of sex (Figure 1).

### Correlation of peripheral blood white blood cell subsets with age

**Figure 2.**
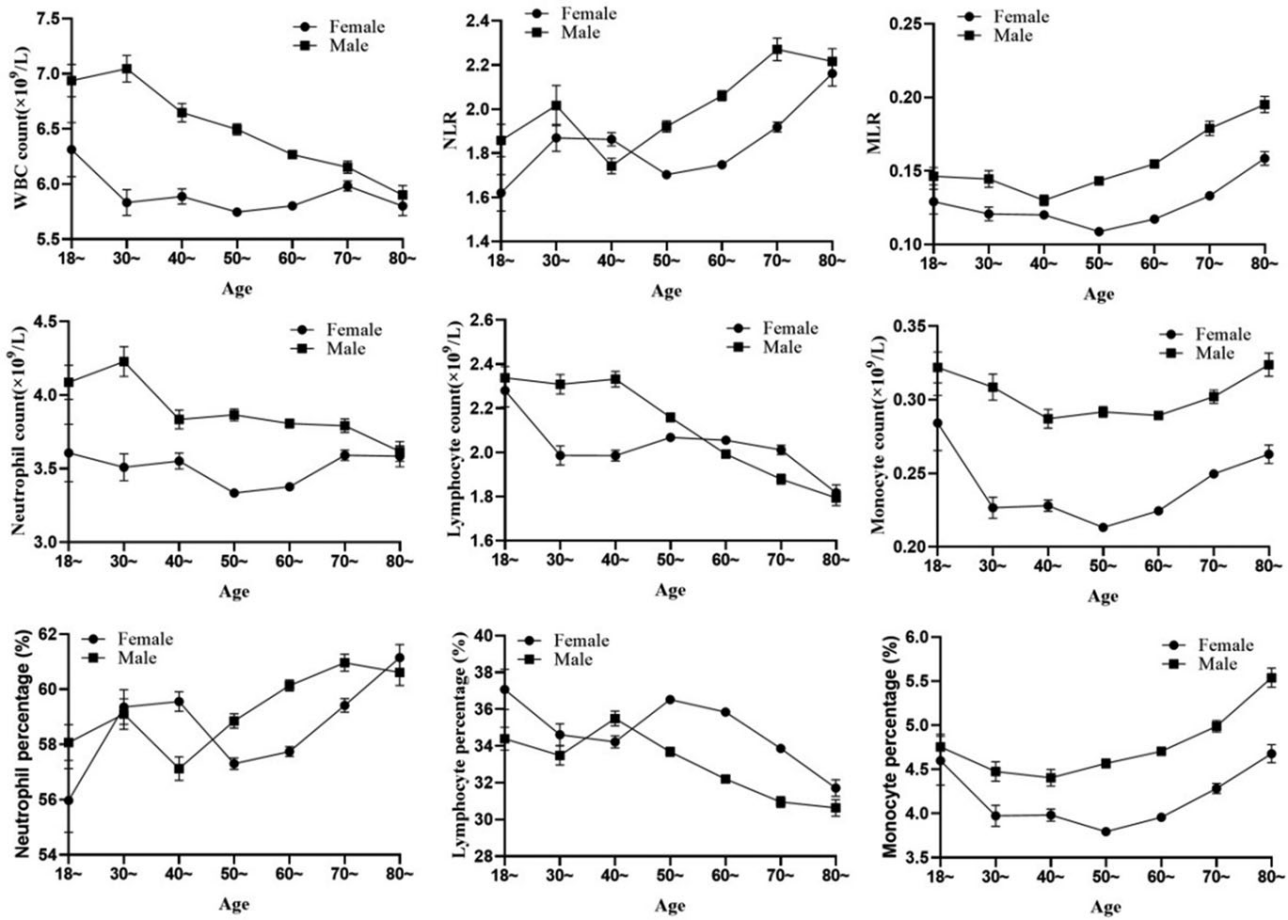
Correlation of subsets of peripheral leukocyte with age.

We observed that the white blood cell counts significantly decreased with age (*F* = 96.45, *P* trend <0.001), but the trend was not significant in females (*F* = 3.448, *P* trend <0.063). The counts and percentages of neutrophils and lymphocytes fluctuated amongst different age groups. The monocyte count and percentage in males were higher than those in females in all age groups. However, the age-associated trend was not linear. These NLR data were not characteristic of age or sex specificity (Figure 2).

### NLR and MLR in patients with hypertension, diabetes, or stroke

We compared the NLR and MLR between subjects with and without chronic disease such as hypertension, diabetes, and stroke. The NLR was higher in both females and males in the hypertension, but was higher in the female group with diabetes. The MLR was also higher in hypertensive subjects than in those without this disease; however, the difference between the MLR values of the two groups was not large. Significant differences in all of these indexes were not observed between the people with or without stroke (Table 2).

### Utility of NLR and MLR as risk biomarkers for hypertension and diabetes

**Figure 3.**
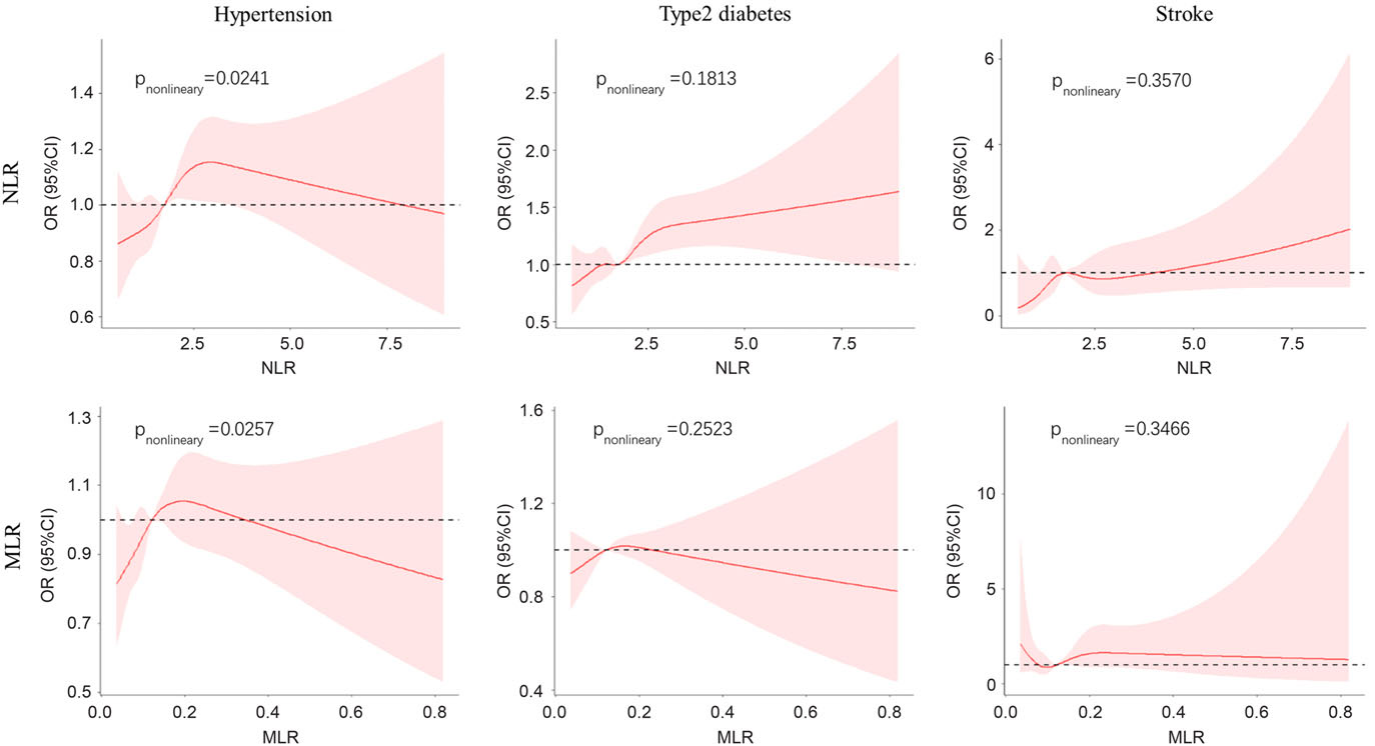
Restricted cubic spline plot of the risk of chronic diseases according to NLR and MLR. Odd ratios (OR) were adjusted for age, sex, and BMI.

The results from logistic regression models showed that the NLR and MLR were associated with chronic diseases such as hypertension and diabetes after adjustment for age, sex, and BMI. As shown in Table 3, an elevated NLR was associated with the risk of hypertension, and the risk for patients in the top quartile of NLR was 1.19-fold higher (95% CI 1.05, 1.35) than for patients in the lowest quartile, while a high MLR also showed a correlation with an increased risk of hypertension in the lowest quartile (OR = 1.15, 95% CI 1.03, 1.29 for the second quartile). The NLR in the fourth quartile was associated with a statistically significantly increased risk of diabetes compared with the first quartile (OR = 1.40, 95% CI 1.19, 1.66). No factors except for age were significantly associated with the risk of stroke. The does–response relationship between NLR, MLR and chronic diseases risk was analysed by restricted cubic spline model. The results showed NLR and MLR were associated with risk of hypertension (*P*_nonlinearity_ =0.0241 for NLR, *P*_nonlinearity_ = 0.0257 for MLR, respectively.) There was no non-linearity significance dose–response relationship of NLR, MLR with diabetes and stroke (Figure 3).

**Table 1.** Characteristics of participants in the present study

*Variables*	*Male*	*%/SD*	*Female*	*%/SD*	*Total*	*%/SD*
No.	4768	45.134	5796	54.866	10 564	100
Age						
<60 years	1811	37.98	2152	37.13	3963	37.514
≥60 years	2957	62.02	3644	62.87	6601	62.486
BMI						
<18.5	68	1.43	133	2.29	201	1.903
18.5˜	1669	35.00	2486	42.89	4155	39.332
24.0˜	2696	56.54	2741	47.29	5437	51.467
30˜	325	6.82	420	7.25	745	7.052
Missing	10	0.21	16	0.28	26	0.246
Hypertension						
Presence	2207	46.29	2794	48.21	5001	47.340
Non-presence	2561	53.71	3002	51.79	5563	52.660
Diabetes						
Presence	773	16.21	944	16.29	1717	16.253
Non-presence	3995	83.79	4852	83.71	8847	83.747
Stroke						
Presence	52	1.09	42	0.72	94	0.890
Non-presence	4716	98.91	5754	99.28	10 470	99.110
WBC Subset						
WBC	6.37	1.64	5.83	1.46	6.07	1.57
Neutrophil	3.84	1.29	3.44	1.14	3.62	1.22
Lymphocyte	2.05	0.65	2.03	0.61	2.04	0.63
Monocyte	0.30	0.12	0.23	0.09	0.26	0.11
NLR	2.043	1.059	1.807	0.755	1.913	0.912
MLR	0.157	0.091	0.121	0.076	0.137	0.085

**Table 2.** Levels of NLR, MLR in participants with and without specific-complication

*Diseases*	*Female*			*Male*		
	*Without*	*With*	*P-value*	*Without*	*With*	*P-value*
Hypertension						
NLR	1.67 (1.29, 2.09)	1.70 (1.34, 2.15)	0.001	1.80 (1.41, 2.34)	1.88 (1.45, 2.44)	0.001
MLR	0.11 (0.08, 0.14)	0.11 (0.08, 0.15)	<0.001	0.14 (0.10, 0.18)	0.14 (0.11, 0.19)	0.016
Diabetes						
NLR	1.64 (1.29, 2.09)	1.78 (1.37, 2.26)	<0.001	1.83 (1.43, 2.38)	1.88 (1.42, 2.53)	0.179
MLR	0.11 (0.08, 0.14)	0.11 (0.08, 0.15)	0.021	0.14 (0.10, 0.19)	0.14 (0.11, 0.19)	0.887
Stroke						
NLR	1.67 (1.31, 2.12)	1.76 (1.48, 2.30)	0.114	1.84 (1.43, 2.39)	1.79 (1.60, 2.85)	0.191
MLR	0.11 (0.08, 0.15)	0.13 (0.09, 0.17)	0.045	0.14 (0.11, 0.19)	0.15 (0.10, 0.20)	0.558

**Table 3.** Estimation the risk of hypertension, diabetes and stroke exposure in different levels of NLR and MLR

*Factors*	*No.*	*Hypertension*			*Diabetes*			*Stroke*		
		*OR*	*95% CI*	*P-value*	*OR*	*95% CI*	*P-value*	*OR*	*95% CI*	*P-value*
Age(≥60/<60)	3943/6437	1.82	1.67, 1.98	<0.001	1.55	1.39, 1.74	0.00	1.85	1.14, 3.00	0.01
Sex(M/F)	4678/5702	0.85	0.78, 0.93	<0.001	0.95	0.85, 1.06	0.34	1.38	0.89, 2.14	0.15
BMI				<0.001^*^			<0.001^*^			0.532^*^
<18.5	198	1.00	reference		1	reference		1	reference	
18.5˜	4092	2.01	1.44, 2.8	<0.001	2.87	1.51, 5.46	<0.001	1.85	0.25, 13.59	0.54
24˜	5357	3.52	2.53, 4.91	<0.001	4.30	2.27, 8.18	<0.001	1.77	0.24, 12.92	0.58
30˜	733	5.33	3.72, 7.64	<0.001	5.70	2.94, 11.03	<0.001	0.82	0.08, 7.91	0.86
NLR				0.003^*^			<0.001^*^			0.342^*^
<1.36	2590	1	reference		1	reference		1	reference	
1.36˜	2595	1.08	0.96, 1.21	0.20	1.03	0.88, 1.2	0.74	2.33	1.18, 4.61	0.02
1.74˜	2597	1.14	1.02, 1.29	0.02	1.15	0.98, 1.35	0.08	1.60	0.76, 3.36	0.21
2.25˜	2598	1.19	1.05, 1.35	0.01	1.40	1.19, 1.66	<0.001	1.98	0.94, 4.18	0.07
MLR				0.183^*^			0.335^*^			0.534^*^
<0.089	2580	1	reference		1	reference		1	reference	
0.089˜	2583	1.15	1.03, 1.29	0.01	0.91	0.78, 1.06	0.22	0.48	0.23, 1.00	0.05
0.121˜	2575	1.10	0.98, 1.24	0.12	0.88	0.75, 1.04	0.13	0.92	0.49, 1.70	0.78
0.164˜	2642	1.13	0.99, 1.28	0.07	0.92	0.77, 1.09	0.32	1.10	0.58, 2.10	0.78

* Asterisk stands for *P*-value for trend

## Discussion

In the present study, we retrospectively analysed the distribution of peripheral blood white blood cell subsets in a community population. The whole blood parameters from 10 564 adults showed that WBC counts were higher in males than in females (6.36 ± 1.63 × 10^9^ in males versus 5.83 ± 1.46 × 10^9^ in females). No significant difference in the white blood cell count was observed between sexes and the white blood cell count was slightly higher in women amongst the adult heathy population in previous studies (Elderdery & Alshaiban, 2017; Fondoh *et al.*, 2020). The reasons why our results were inconsistent with previous reports were potentially attributed to the subjects recruited in this study. All participants in our study were older, and some had chronic diseases. As shown in the studies by Jakob Zierk *et al.* (Zierk *et al.*, 2020) and Yang *et al.* (Yang *et al.*, 2020), the leukocyte count was higher in males older than 50 years of age but lower in younger males, and the number of white blood cells increased in individuals with metabolic syndrome. The counts of leukocyte subsets, such as neutrophils, lymphocytes, and monocytes, increased with the BMI; however, the percentages of neutrophils and monocytes were inversely associated with BMI. In contrast, the percentage of lymphocytes was higher in individuals with a higher BMI. As a unique WBC subset, the lymphocyte percentage is higher in females than in males. The peripheral lymphocyte count reflects the capacity of the host immune response to predict the outcome of diseases (Afghahi *et al.*, 2018; Semerano *et al.*, 2019). Inconsistent conclusions have been reported on the difference in the absolute lymphocyte count between sexes. Wongkrajang *et al.* found that the lymphocyte count was higher in males, but the percentage was not different between sexes (Wongkrajang *et al.*, 2018), which was opposite to our finding. Although the differences were not clinically relevant according to the Wongkrajang group, the peripheral lymphocyte percentage more accurately predicted the survival of patients with CRC than the peripheral lymphocyte count (Iseki *et al.*, 2017). This inconsistent conclusion must be confirmed by conducting more studies in various fields.

Peripheral lymphocytes, neutrophils, and monocytes are the main subsets of white blood cells, and they play an important role in the individual’s immune system. This study revealed that white blood cell, neutrophil, and lymphocyte counts and percentages decreased with increasing age, while the monocyte count and percentage increased with ageing. An older age is also associated with a decreasing lymphocyte count (Lehtonen *et al.*, 1990; Rea *et al.*, 1996). Although its mechanism is not very clear, possible explanations may include age-related thymic involution, which leads to a change in the composition of lymphocyte subsets with age (Rea *et al.*, 1996; Qin *et al.*, 2016), and consequently, a change in overall immune competence (Linton & Dorshkind, 2004). With increasing age, the human body becomes relatively frail. The lymphocyte count, a marker of inflammation and immunosuppression, might be useful for identifying frail patients and evaluating people’s health conditions (Nunez *et al.*, 2020).

Because the NLR and MLR are reliable systemic inflammatory markers, we tested their association with three common chronic diseases in a community population. As a result, the mean NLR and MLR in male patients with hypertension or diabetes was significantly greater than that in individuals without these diseases. Meanwhile, the NLR and MLR were elevated in female patients with hypertension, but not diabetes. We identified an association between an elevated NLR and a high risk of hypertension and diabetes, but not a risk of stroke. A dose–response relationship of NLR, MLR with hypertension is shown in a non-linear dose–response manner, but no significant relationship with stroke or diabetes. The MLR in this study was not a perfect indicator of chronic diseases, although a trend of a high MLR was associated with an insignificantly increased risk of hypertension and diabetes. Studies have identified the NLR as a risk indicator for overall and cause-specific mortality, such as cardiovascular disease and stroke (Kim *et al.*, 2018; Fest *et al.*, 2019; Maestrini *et al.*, 2020). Neutrophils were first considered the major arm of the innate immune system. Recently, accumulating evidence has suggested that neutrophils are able to regulate many processes, such as acute injury and repair, cancer, autoimmunity, and chronic inflammation (Liew & Kubes, 2019). Inflammation has been confirmed to be associated with the risk of developing hypertension, diabetes, and stroke (Lontchi-Yimagou *et al.*, 2013; Tsounis *et al.*, 2014; Anrather & Iadecola, 2016). The NLR potentially reflects the balance between innate (i.e., neutrophils) and acquired (i.e., lymphocytes) immune responses in the body (Rucker *et al.*, 2018). Therefore, the NLR is widely confirmed to be a potential biomarker for health conditions. In the present study, the NLR was increased in patients with hypertension and diabetes compared with the common population. Therefore, we postulate that the NLR is useful for screening chronic diseases amongst community residents. The value of the capacity of the MLR to evaluate health conditions requires further study.

### Limitations

This study has unavoidable limitations. First, the sample source were residents of a single community, leading to limited representativeness. Second, the distribution of leukocyte subsets in the 40-year-old or younger group showed an unstable trend, and one potential reason may be that the young persons (38.52% of the total) who were recruited had a lower composition. The causal role of the NLR or MLR in hypertension or diabetes was unable to be determined because the data were obtained from a cross-sectional study. Hence, future studies are needed amongst additional communities that investigate younger participants, as well as cohort studies.

## Conclusions

Based on these findings, leukocyte counts and leukocyte subset counts had different distributions in populations stratified by sex, age, and BMI. In summary, elevated leukocyte counts are mostly present in older, fatter, and male populations. The NLR will be considered as a potential biomarker to evaluate the risk of chronic diseases, especially hypertension and diabetes.
